# The complete mitochondrial genome of *Protobothrops kaulbacki* (Squamata: Viperidae)

**DOI:** 10.1080/23802359.2017.1307703

**Published:** 2017-04-05

**Authors:** Xing Kang, Yu Zhang, Lifu Qian, Ping Sun, Chencheng Wang, Ke Fang, Tao Pan, Baowei Zhang, Dingqi Rao, Hui Wang

**Affiliations:** a School of Life Sciences, Anhui University, Hefei, Anhui, China;; b Kunming Institute of Zoology, Chinese Academy of Sciences, Kunming, Yunnan, China

**Keywords:** *Protobothrops kaulbacki*, mitochondrial genome, phylogentic tree

## Abstract

*Protobothrops kaulbacki* is a new record in China, and in this study, the complete mitochondrial genome sequence of *P. kaulbacki* had been determined. The length of mitogenome is 17,237 bp, including 13 protein-coding genes, 22 tRNA genes, 2 rRNA genes (12S and 16S rRNA), 1 origin of L-strand replication (OL), and 2 control regions (CRs). The maximum-likelihood (ML) tree based on the whole mitogenome shown that *P. kaulbacki* belongs to the genus *Protobothrops*.

Kaulback’s pitviper, *Protobothrops kaulbacki* is a kind of venomous snakes, belonging to the genus *Protobothrops* of family Viperidae. This species was firstly reported by Malcolm A. Smith in 1940, and found in Pangnamadim, north of Burma (Smith 1940). In 2004, Kaulback’s pitviper was collected by biologists during the Herpetological survey, and this was a new record in China (Rao & Zhao 2005). Therefore, nine species are currently recognized in China within the genus *Protobothrops*: *P. maolanensis*, *P. jerdonii*, *P. xiangchengsis*, *P. cornutus*, *P. kaulbacki*, *P. mangshanensis, P. mucrosquamatus*, *P. himalayanus*, and *P. dabieshanensis* (Yang et al. 2011; Huang et al. 2012; Pan et al. 2013). Because of less information for this genus, we need more molecular data and genome research for the *P. kaulbacki*.

The *P. kaulbacki* samples were collected in Mêdog County of the Southern Xizang, China (N 29°19′, E 95°10′) in August 2004. Presently, the specimen was stored in the Museum of Shenyang Normal University (Rao & Zhao 2005). We chose muscle tissue to extract the whole genomic DNA by a standard proteinase-K/phenol–chloroform protocol (Sambrook & Russell 2006). The entire mitogenome was amplified by polymerase chain reaction (PCR) using 20 primers. Here, we sequenced the complete mitochondrial genome of *P. kaulbacki* and deposited in GenBank (Accession number KY695463).

The complete mtDNA of *P. kaulbacki* is 17,237 bp in length, and the gene order was identical to *P. dabieshanensis* (Huang et al. 2012), including 13 protein-coding genes, 22 tRNA genes, 2 rRNA genes (12S and 16S rRNA), 1 origin of L-strand replication (OL), and 2 control regions (CRs). The base composition of the mitogenome was 33.3% A, 29.7% C, 12.3% G, and 24.7% T. The *ND6* subunit gene, OL, and eight tRNA genes (*tRNA^Pr^°*, *tRNA^Gln,^ tRNA^Ala^*, *tRNA^Asn^*, *tRNA^Cys^*, *tRNA^Tyr^*, *tRNA^Ser^*^(UCN)^, and *tRNA^Glu)^*) were encoded on the L-strand, the remaining genes were encoded on the H-strand. The control region1 (CR1) of the *P. kaulbacki* mtDNA is 1043 bp long and lies between the *tRNA^Thr^* and *tRNA^Phe^* genes, while the control region2 (CR2) is 1067 bp long, between the *tRNA^Pr^°* and *tRNA^Leu^*^(UUR)^ genes. The origin of L-strand replication (OL) in *P. kaulbacki* was inside a cluster of five tRNA genes (*tRNA^Trp^, tRNA^Ala^*, *tRNA^Asn^*, *tRNA^Cys^*, and *tRNA^Tyr^*), which is 34 bp long, as seen in most vertebrates.

The maximum-likelihood (ML) tree were generated with RAxML 7.0.3 (Stamatakis 2008) based on the whole mitogenome, *Achalinus meiguensis* was selected as an outgroup. In this process, the optimal substitution model (GTR) was implemented via JModelTest 2 (Darriba et al. 2012). As shown in Figure 1, the phylogenetic tree were split into two well-supported major clades, the *Protobothrops* and the *Gloydius*, what’s more, *P. kaulbacki* is closer to *P*. *himalayanus*, belong to the genus *Protobothrops*.

**Figure 1.**
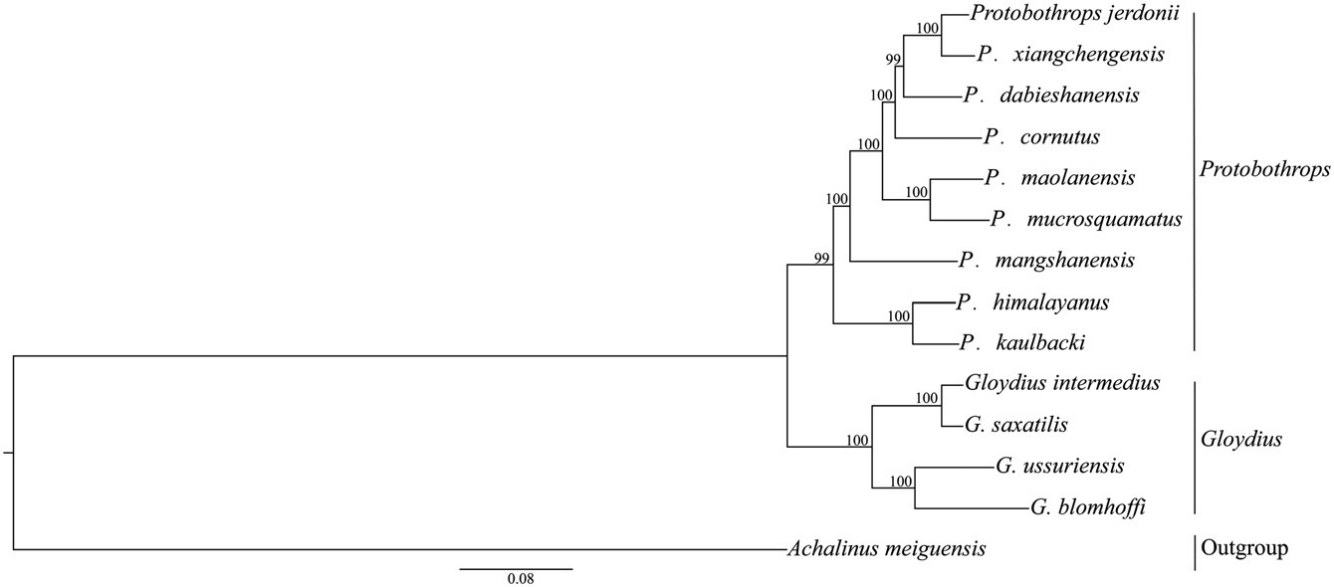
The maximum-likelihood (ML) tree based on the whole mitogenome. Numbers at the nodes are bootstrap values of the ML analysis. The GenBank accession number of species are *P. jerdonii* (NC_021402), *P. xiangchengensis* (KF460436), *P. dabieshanensis* (KF003004), *P. cornutus* (NC_022695), *P. maolanensis* (NC_026051), *P. mucrosquamatus* (KT447436), *P. mangshanensis* (NC_026052), *P. himalayanus* (NC_029165), *P. kaulbacki* (KY695463), *G. intermedius* (NC_025560), *G. saxatilis* (NC_025666), *G. ussuriensis* (NC_026553), *G. blomhoffi* (NC_011390), *A. meiguensis* (NC_011576).

Because of the unique characteristics of mitochondrial DNA, mitochondrial genome sequences have been proven to play a significant role in phylogenetic relationships. We expect the present result to provide fundamental resources during analyzing the phylogenetic relationship within *P. kaulbacki* and other species in Viperidae.
